# Supplementary figures and images for: Morphine self-administration is inhibited by the antioxidant N‐acetylcysteine and the anti-inflammatory ibudilast; an effect enhanced by their co-administration
Source: PLoS One. 2024 Oct 29;19(10):e0312828. doi: 10.1371/journal.pone.0312828 (PMC11521314; doi:10.1371/journal.pone.0312828)

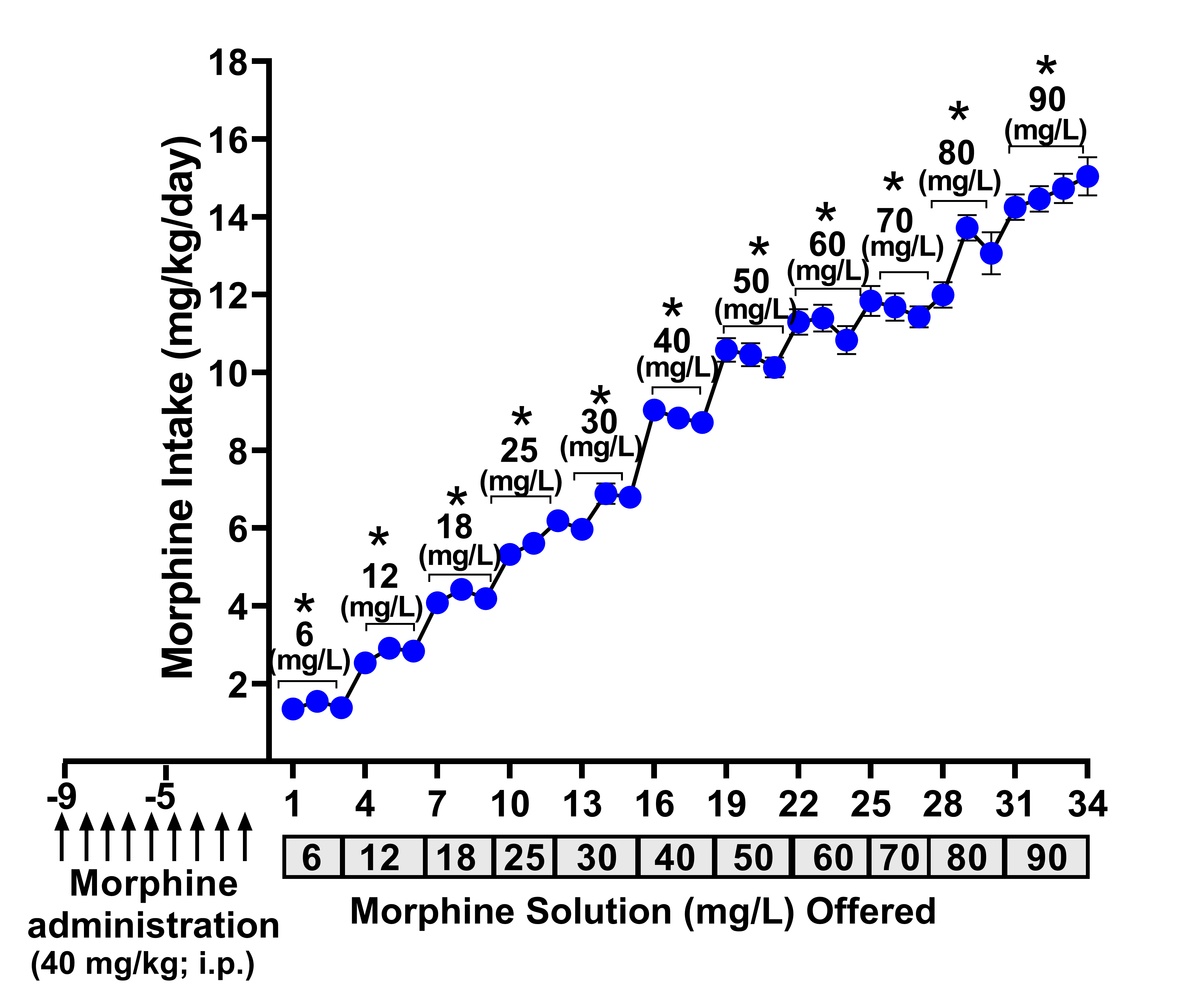

Supplement: S1 Fig — Figure shows morphine consumption expressed as mg of morphine consumed per kilogram of body weight per day (mean ± SEM) of rats given free choice between a morphine sulfate solution of increasing concentration and water 24 hours/day (n = 24). Arrows indicate intraperitoneal administration of morphine. Two-way ANOVA (concentration × day) of all voluntary oral morphine intake data indicated a significant effect of the concentration of the morphine solution offered to rats [Fconcentration(10,253) = 351.5, p<0.0001], day [Fday(1548,400.8) = 11.61, p<0.0001] and concentration × day interaction [Finteraction(20,506) = 4.731, p<0.0001]. Tukey’s post hoc test revealed that each increase in morphine concentration in the range of 6 mg/L to 90 mg/L resulted in a significant increase (****p<0.0001) in daily morphine intake. (TIF) [file pone.0312828.s001.tif]

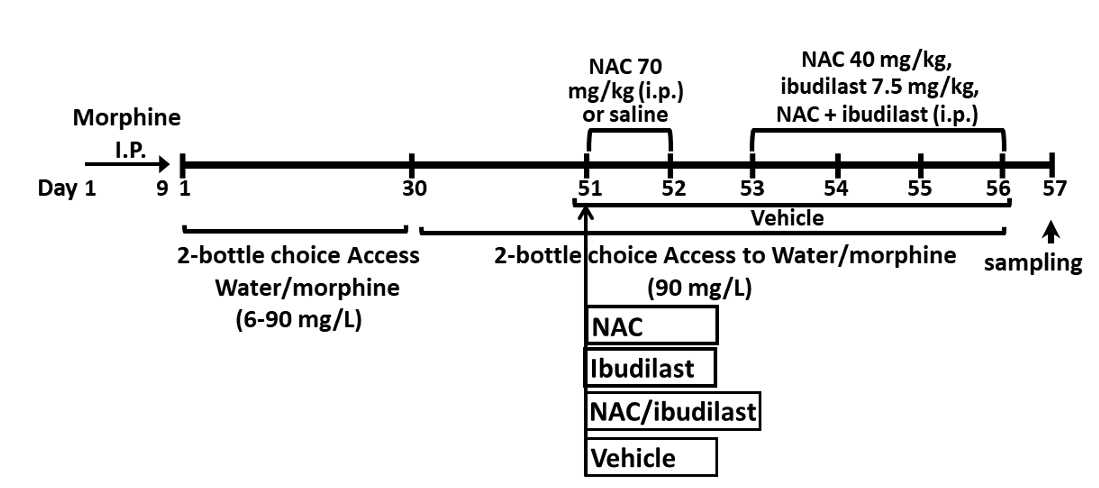

Supplement: S2 Fig — The timeline outlines the following events performed on the rats: (i) Intraperitoneal administration of morphine; (ii) The option to choose between oral morphine and water; (iii) On day 51, rats voluntarily drinking morphine received intraperitoneal loading doses of NAC or saline; (iv) On day 53, rats continuing to drink morphine and received intraperitoneal maintenance doses of vehicle, NAC, Ibudilast, or NAC + ibudilast; (v) On day 57, brain tissue samples were collected. (TIF) [file pone.0312828.s002.tif]

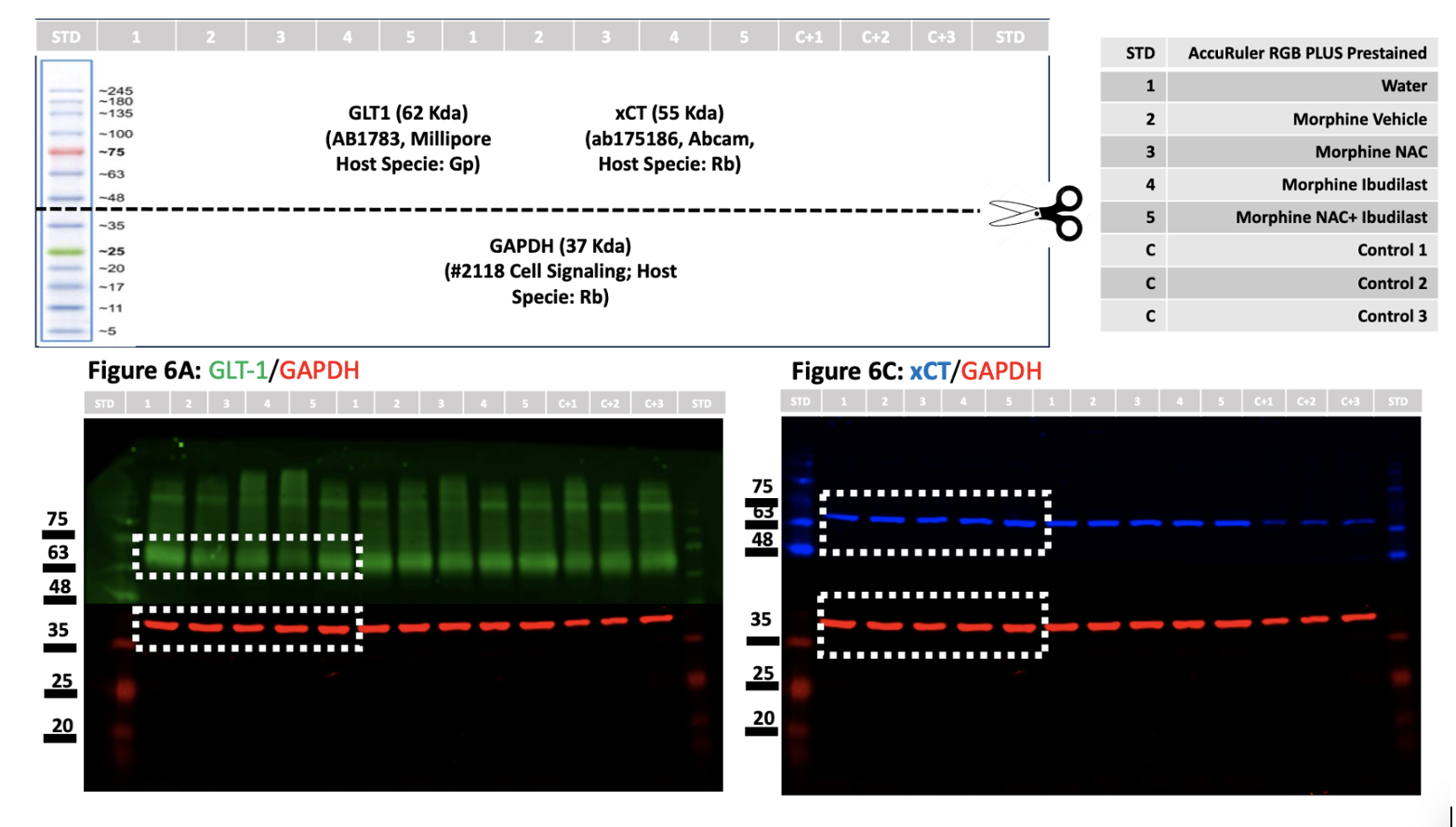

Supplement: S3 Fig — The lanes of the unedited blots that appear in the cropped images in the manuscript are highlighted. (TIF) [file pone.0312828.s003.tif]
