# Supplementary material for: Morphine self-administration is inhibited by the antioxidant N‐acetylcysteine and the anti-inflammatory ibudilast; an effect enhanced by their co-administration
Source: PLoS One. 2024 Oct 29;19(10):e0312828. doi: 10.1371/journal.pone.0312828 (PMC11521314; doi:10.1371/journal.pone.0312828)
Supplement: Attachment — Submitted filename: Reply to Reviewers, Quintanilla et al PLOS ONE reply 2.docx [file pone.0312828.s005.docx]

October 5th, 2024

Dr. Shao-Jun Tang

Academic Editor

PLOS ONE

Dear Dr. Tang:

*Re: ID PONE-D-24-20382R1: Final Decision Being Processed -*

Thank you for informing us that our manuscript entitled “Morphine self-administration is inhibited by the antioxidant N-acetylcysteine and the anti-inflammatory Ibudilast; an effect enhanced by their coadministration” by María Elena Quintanilla, Paola Morales, Daniela Santapau, Javiera Gallardo, Rocio Rebolledo, Gabriel Rivas, Tirso Acuña, Mario Herrera-Marschitz, Yedy Israel and Fernando Ezquer has been judged scientifically suitable for publication in PLOS ONE, following the correction of minor comments by Reviewer 2.

We have addressed the minor comments provided by reviewer 2 and have modified our manuscript accordingly. Please find below a reply organized as Reviewer’s Comment; Reply and Modifications. The modifications have been incorporated into the resubmitted manuscript (clean copy), and the changes are highlighted in red in the marked-up copy.

We hope that the recommended changes incorporated on our manuscript will make this revised version acceptable for publication in PLOS ONE.

Sincerely,

Fernando Ezquer, PhD

Center for Regenerative Medicine

School of Medicine

Universidad del Desarrollo

Chile

**Point-by-point ReplIES and Modifications**

**Reviewer #2**

***Rev#2-Comment 1***: Authors appropriately addressed my major points. I have only few remaining minor comments to make***.*** Line 230: Authors specified that animals were euthanized one hour after the last recorded morphine intake, it remains unclear however whether morphine was withdrawn during that hour (i.e. rats had one hour of abstinence) or not. This is a crucial information for the reproducibility of the study, and I warmly recommend to clarify it.

***Rev#2 Reply and Modification 1***: Thank you very much for this important comment. Now we clarify (line 230): "Seventeen hours after the last administration of NAC and ibudilast, and one hour after the last recorded morphine intake, rats that continued to have access to the morphine solution and water were anesthetized with a cocktail of 60 mg/kg ketamine HCl, 10 mg/kg xylazine HCl and 4 mg/kg acepromazine (administered intramuscularly in a volume of 1.9 mL/kg) [43], intracardially perfused with 100 mL of 0.1 M PBS (pH 7.4) and euthanized”.

***Rev#2-Comment 2:*** Line 314 and Fig 1: when ANOVA finds no significant effects, it does not require to be followed by post-hoc tests. This apply also to other sessions of the manuscript where a non-significant effect is reported.

***Rev#2 Reply and Modification 2.*** Thank you very much for your comment. We have now removed mentions of post-hoc tests in the following locations of the Results section: lines 314, 349, and 351, as the ANOVAs did not find any significant differences.
